# Inhibiting phosphorylation of the oncogenic PAX3-FOXO1 reduces alveolar rhabdomyosarcoma phenotypes identifying novel therapy options

**DOI:** 10.1038/oncsis.2015.2

**Published:** 2015-03-30

**Authors:** J M Loupe, P J Miller, D R Ruffin, M W Stark, A D Hollenbach

**Affiliations:** 1 Louisiana State University Health Sciences Center, Department of Genetics, New Orleans, LA, USA; 2 Department of Pathology, Children's Hospital, New Orleans, LA, USA

## Abstract

Patients with translocation-positive alveolar rhabdomyosarcoma (ARMS), an aggressive childhood tumor primarily characterized by the PAX3-FOXO1 oncogenic fusion protein, have a poor prognosis because of lack of therapies that specifically target ARMS tumors. This fact highlights the need for novel pharmaceutical interventions. Posttranslational modifications such as phosphorylation are becoming attractive biological targets for the development of such interventions. Along these lines, we demonstrated that PAX3-FOXO1 is phosphorylated at three specific sites and that its pattern of phosphorylation is altered relative to wild-type Pax3 throughout early myogenesis and in ARMS tumor cells. However, little work has been performed examining the effect of directly inhibiting phosphorylation at these sites on ARMS development. To address this gap in knowledge, we used small molecule inhibitors or mutational analysis to specifically inhibit phosphorylation of PAX3-FOXO1 to investigate how altering phosphorylation of the oncogenic fusion protein affects ARMS phenotypes. We found that inhibiting the phosphorylation of PAX3-FOXO1 at Ser201 significantly reduced migration, invasion and proliferation in two independent ARMS tumor cell lines. Further, we found that inhibition of phosphorylation at Ser205 also decreased proliferation and anchorage-independent growth. Consistent with these *in vitro* results, we demonstrate for the first time that PAX3-FOXO1 is phosphorylated at Ser201 and Ser205 in a primary tumor sample and in tumor cells actively invading the surrounding normal tissue. This report is the first to demonstrate that the direct inhibition of PAX3-FOXO1 phosphorylation reduces ARMS tumor phenotypes *in vitro* and that these phosphorylation events are present in primary human ARMS tumors and invading tumor cells. These results identify phosphorylation of PAX3-FOXO1, especially at Ser201, as a novel biological target that can be explored as a promising avenue for ARMS therapies.

## Introduction

Rhabdomyosarcoma (RMS), one of the most common solid tumors in children,^1^ is comprised of two main histological subtypes: embryonal and alveolar (ARMS). ARMS, the more aggressive subtype, is primarily defined by the t(2;13)(q35; q14) translocation, which fuses the amino-terminal region of Pax3 to the carboxyl-terminal sequences of FOXO1.^2, 3, 4^ The resulting PAX3-FOXO1 oncogenic fusion protein has altered molecular activities relative to wild-type Pax3,^5, 6, 7, 8, 9, 10^ which are believed to contribute to ARMS tumor phenotypes.^11^ Patients diagnosed with PAX3-FOXO1-positive ARMS have a 4-year survival rate of 8%^12^ which stems from the chemoresistance of metastatic tumors combined with a current lack of effective therapies specific for targeting ARMS. This information highlights the necessity of understanding the underlying biological and biochemical processes that contribute to the genesis of ARMS to develop much needed therapeutic alternatives.

Posttranslational modifications such as phosphorylation are common mechanisms for the regulation of transcription factors. As such, inhibition of these phosphorylation events provides an attractive target for drug development.^13, 14^ We published that wild-type Pax3 is phosphorylated at Ser201 and Ser205 by the kinases GSK3β and CK2, respectively.^15, 16^ Upon the induction of differentiation, phosphorylation at Ser201 persists. However, phosphorylation at Ser205 is rapidly lost with a concomitant increase in phosphorylation on Ser209, again mediated by CK2.^16, 17^ In contrast, we found that PAX3-FOXO1 is phosphorylated on Ser201 and Ser205 during proliferation; this status remains unaltered throughout myogenesis with no increase in phosphorylation at Ser209.^15, 16^ Therefore, the aberrant phosphorylation of PAX3-FOXO1 may affect normal myogenesis to contribute the development of ARMS.

Previous work demonstrated that inhibiting phosphorylation of PAX3-FOXO1 in T-antigen-transformed human embryonic kidney cells (293T cells), a non-physiologically relevant *in vitro* cellular model,^18^ altered its transcriptional activity. Others demonstrated that small molecule inhibitors of GSK3β affected the viability and transformation capabilities of an ARMS tumor cell line.^19^ However, the first study utilized a general mutation approach that altered several serine residues within a region without specifically targeting the known sites, whereas the second study failed to demonstrate that the small molecule inhibitors directly altered phosphorylation of PAX3-FOXO1. Further, neither of these studies demonstrated a direct dependence of biological outcomes on alterations of the specific and identified PAX3-FOXO1 phosphorylation events. Finally, PAX3-FOXO1 phosphorylation has yet to be studied in human primary ARMS tumor samples. Therefore, we wished to determine how inhibiting specific sites of PAX3-FOXO1 phosphorylation affects known ARMS tumor phenotypes and how these biological effects correlate to primary tumor samples to identify potential ARMS-specific biological targets for future therapy development.

In this study, we utilize small molecule inhibitors of GSK3β or phospho-incompetent mutations that individually target the known sites of phosphorylation to determine how inhibiting these events on PAX3-FOXO1 affects ARMS tumor phenotypes. Our results demonstrate that inhibitors of GSK3β reduce phosphorylation of PAX3-FOXO1 at Ser201 and that inhibition of this event, either through small molecule inhibitors or mutational analysis reduces migration, invasion and proliferation in two independent ARMS tumor cell lines. Further, inhibition of phosphorylation at Ser205 by mutational analysis reduces ARMS tumor cell proliferation and anchorage-independent growth. Finally, we demonstrate that phosphorylation of PAX3-FOXO1 at Ser201 and Ser205 are present in human primary ARMS tumors and in cells that infiltrate the surrounding normal tissue. Taken together, our results support the idea that phosphorylation of PAX3-FOXO1 is an important contributor to ARMS tumor phenotypes and as such could be a key biological target for ARMS therapy development.

## Results

### GSK3β inhibitors reduce phosphorylation of endogenous PAX3-FOXO1 at Ser201

**Figure 1:**
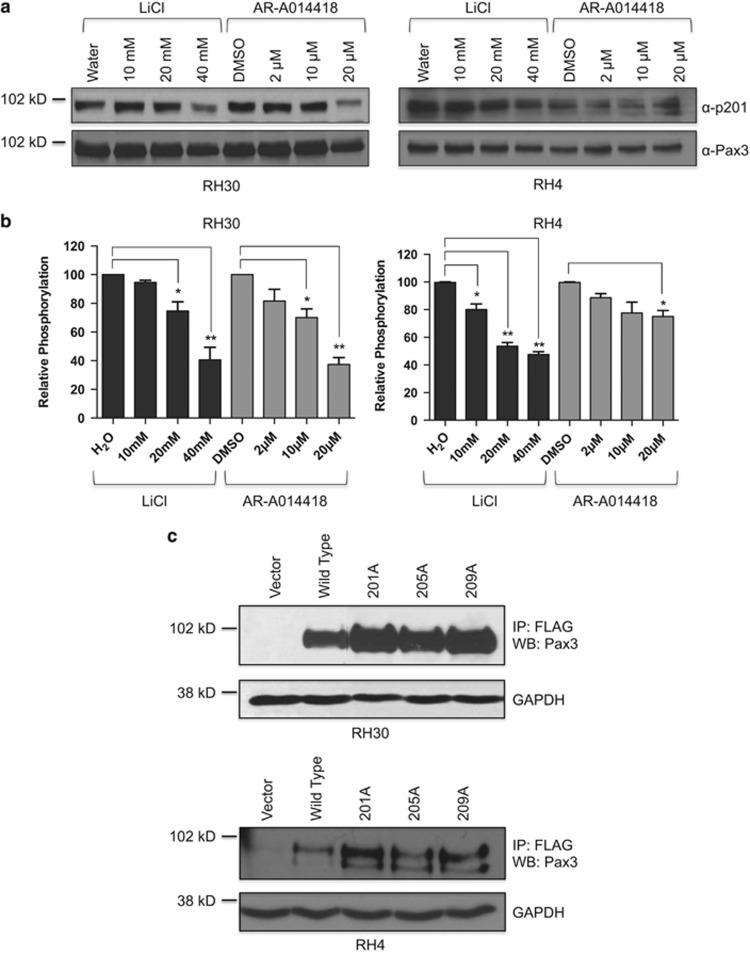
Expression and phosphorylation of endogenous PAX3-FOXO1 or PAX3-FOXO1 phosphomutants. (**a**) Total extracts were made from RH30 (left panel) or RH4 (right panel) ARMS tumor cells treated with increasing concentrations of LiCl or AR-A014418. The presence of endogenous PAX3-FOXO1 (bottom panel) or endogenous PAX3-FOXO1 phosphorylated at Ser201 (top panel) was determined by western blot analysis on 25 μg of total cell extract using an antibody specific for PAX3 or the Ser201 phospho-specific antibody. (**b**) Phospho-PAX3-FOXO1 and total PAX3-FOXO1 were quantified by densitometry after which phospho-PAX3-FOXO1 was normalized for total PAX3-FOXO1. Results are plotted as relative phosphorylation with non-treated cells being given a value of 100. Error bars represent the standard deviation from three independent determinations and *P*-values were computed using non-parametric two-way analyses of variance comparing each treatment condition to results with non-treated cells. (*P=0.03, **P=0.004). (**c**) Total cell extracts were made from RH30 (top panels) or RH4 (bottom panels) cells stably transduced with vector only (vector), wild-type PAX3-FOXO1 (WT) or the indicated PAX3-FOXO1 phospho-mutant. The presence of ectopically expressed PAX3-FOXO1 was determined by immunoprecipitation-western blot analysis, as described in the Materials and Methods.

Others demonstrated that small molecule inhibitors of GSK3β affect the proliferation and viability of ARMS tumor cells.^19^ We determined that GSK3β phosphorylates PAX3-FOXO1 at Ser201 in primary myoblasts and in ARMS cell lines.^16^ However, no studies correlated the inhibition of GSK3β with a decrease in phosphorylation at Ser201 or how these changes affect ARMS tumor phenotypes. Therefore, we incubated the ARMS tumor cell lines RH30 or RH4 with increasing concentrations of the commonly used GSK3β inhibitor lithium chloride (LiCl; IC50=10 mM)^20^ or the highly specific inhibitor AR-A014418 (IC50=10 μM).^21^ We utilized concentrations commonly used in published reports and that were demonstrated to be effective at inhibiting GSK3β *in vivo.*^21, 22, 23^ We determined the level of phosphorylation at Ser201 on endogenous PAX3-FOXO1 by western blot analysis using our anti-Pax3(pSer201) antibody.^16^ After normalization for total PAX3-FOXO1, we observed a titratable decrease in phosphorylation at Ser201 with increasing concentrations of both inhibitors in both cell lines (Figures 1a and b).

Even though we observed titratable inhibition of PAX3-FOXO1 phosphorylation at Ser201, inhibition of GSK3β activity may affect unrelated pathways, as was previously demonstrated in myogenesis and embryonal rhabdomyosarcoma.^23, 24^ Further, inhibition of CK2, a ubiquitous and essential multifunctional enzyme, resulted in non-specific cell death (data not shown) preventing use of these small molecule inhibitors in these studies. Therefore, to determine how specifically inhibiting PAX3-FOXO1 phosphorylation affects ARMS tumor phenotypes we utilized mutants in which each individual site was mutated to a phospho-incompetent alanine (S201A, S205A or S209A). The two ARMS cell lines were stably transduced, selected with puromycin, and selected cells were harvested from three independent transductions and pooled resulting in a single population for each individual mutant. By utilizing a population of transduced cells, we remove the potential for variability that may occur from clonal effects. An immunoprecipitation-western blot analysis demonstrated the ectopic expression of all mutants, with lower levels of expression being seen in the RH4 tumor cell line (Figure 1c). Independent western blot analyses determined that both lines had physiologically relevant levels of ectopic expression that were equivalent to levels of endogenous PAX3-FOXO1 (data not shown).

### Inhibition of PAX3-FOXO1 phosphorylation at Ser201 reduces the migratory ability of ARMS tumor cell lines

**Figure 2:**
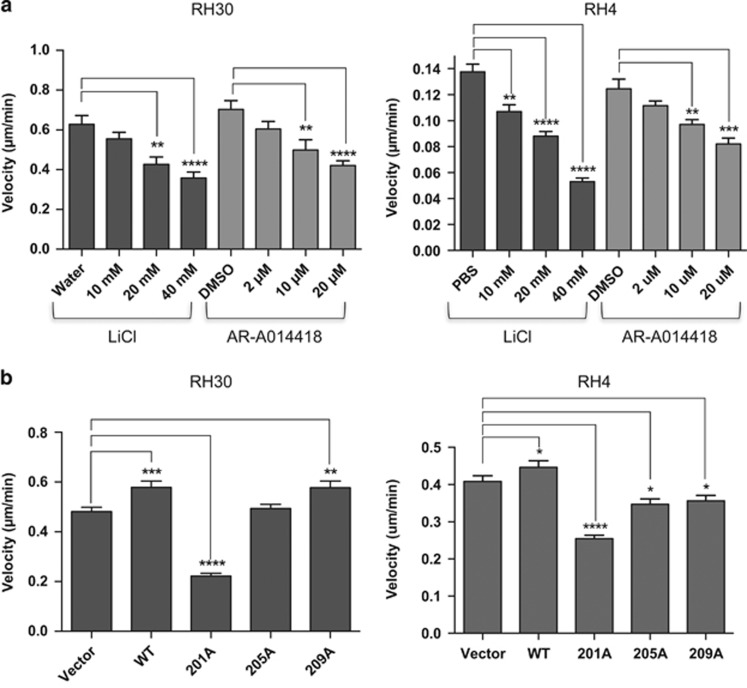
Effect of inhibiting PAX3-FOXO1 phosphorylation on ARMS tumor cell migration. (**a**) ARMS tumor cells RH30 or RH4 were treated with increasing concentrations of LiCl or AR-A014418 or (**b**) stably transduced with empty vector (vector), wild-type PAX3-FOXO1 (WT) or the indicated PAX3-FOXO1 phosphomutants, as described in the Materials and Methods. A scratch was introduced and migration into the wound was monitored by time-lapse microscopy. Individual cells were tracked and their velocities were determined using ImageJ software. For both panels, results are presented as velocity with error bars representing the standard deviation from 60 to 80 independent determinations. *P*-values were computed using non-parametric two-way analyses of variance comparing each treatment condition to results with non-treated cells (**a**) or to the empty vector transduced negative control (**b**). (*P=0.07, **P=0.003, ***P=0.0001 ****P<0.0001).

Cellular migration is an important contributor to tumor development. To determine how inhibition of PAX3-FOXO1 phosphorylation affects ARMS tumor cell migratory ability, we performed wound-healing assays in the presence of GSK3β inhibitors or with cells stably expressing PAX3-FOXO1 phosphomutants. We observed a titratable decrease in migratory ability, as determined by the velocity of movement of individual cells, with increasing concentrations of LiCl or AR-A014418 in both cell lines (Figure 2a). Further, the decreases in migratory ability correlate with our observed decreases in phosphorylation of PAX3-FOXO1 at Ser201 (compare results in Figure 1b and Figure 2a). The ectopic expression of wild-type PAX3-FOXO1, or PAX3-FOXO1 phospho-incompetent at Ser205 or Ser209, had no effect on the migratory ability of RH30 or RH4 cells. In contrast, the stable expression of PAX3-FOXO1 phospho-incompetent at Ser201 inhibited the wound-healing capacity of RH30 and RH4 cells nearly fourfold and twofold, respectively (Figure 2b). These changes in migration did not result from altered proliferation rates as the assays were carried out for 6 h, which is significantly less than the recorded 35–50 h doubling time for these cells (Figure 4), and we did not observe any cellular division while tracking individual cells.

### Inhibition of PAX3-FOXO1 phosphorylation at Ser201 significantly reduces the invasive capacity of ARMS tumor cells

**Figure 3:**
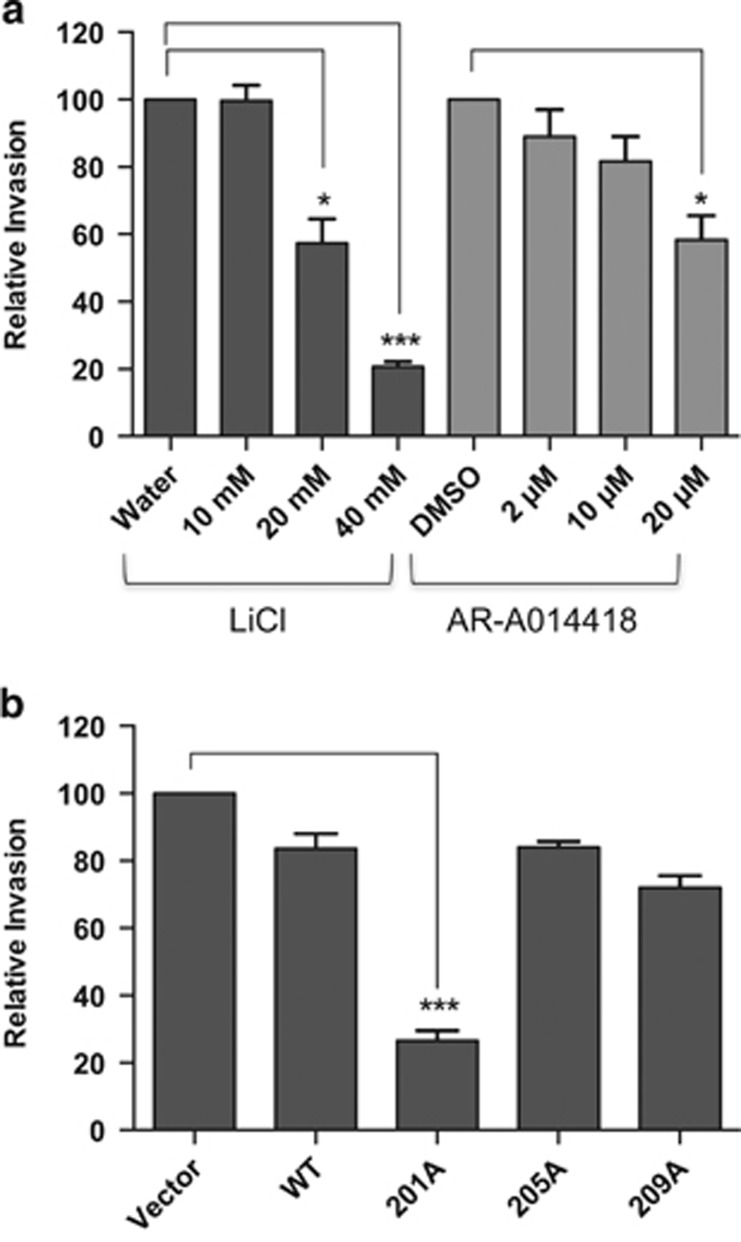
Effect of inhibiting PAX3-FOXO1 phosphorylation on ARMS tumor cell invasion. (**a**) ARMS tumor cells RH30 were treated with increasing concentrations of LiCl or AR-A014418 or (**b**) stably transduced with empty vector (vector), wild-type PAX3-FOXO1 (WT) or the indicated PAX3-FOXO1 phosphomutants, as described in the Materials and Methods. Invasion capacity was determined using the BD Biocoat Tumor Invasion system, a Matrigel-based invasion assay, as described in the Methods. Results are presented as Relative Invasion with non-treated cells (**a**) or empty vector-transduced cells (**b**) being given a relative value of 100. Error bars represent the standard deviation from three independent determinations and *P*-values were computed using non-parametric two-way analyses of variance comparing each treatment condition to results with non-treated cells (**a**) or to the empty vector-transduced negative control (**b**). (*P=0.02, ***P=0.0003).

One of the hallmarks of metastasis is the ability of a tumor cell to invade the basement membrane to initiate movement from the primary tumor site. Therefore, we investigated how inhibition of PAX3-FOXO1 phosphorylation affected the invasive capacity of ARMS tumor cells. RH4 cells do not invade through Matrigel^25^ (and data not shown); therefore, we were only able to utilize RH30 cells for these experiments. We observed a titratable decrease in invasive capacity with increasing concentrations of both LiCl and AR-A014418, resulting in a nearly 80% and 50% decrease, respectively (Figure 3a), again, consistent with the observed decreases of phosphorylation at Ser201. The stable expression of wild-type PAX3-FOXO1, or PAX3-FOXO1 phospho-incompetent at Ser205 or Ser209, had no effect on the invasive capacity of RH30 cells. In contrast, the stable expression of PAX3-FOXO1 phospho-incompetent at Ser201 decreased invasion by nearly 80%, similar to reductions seen in the presence of LiCl (Figure 3b).

### Inhibition of PAX3-FOXO1 phosphorylation reduces proliferation of ARMS tumor cells

**Figure 4:**
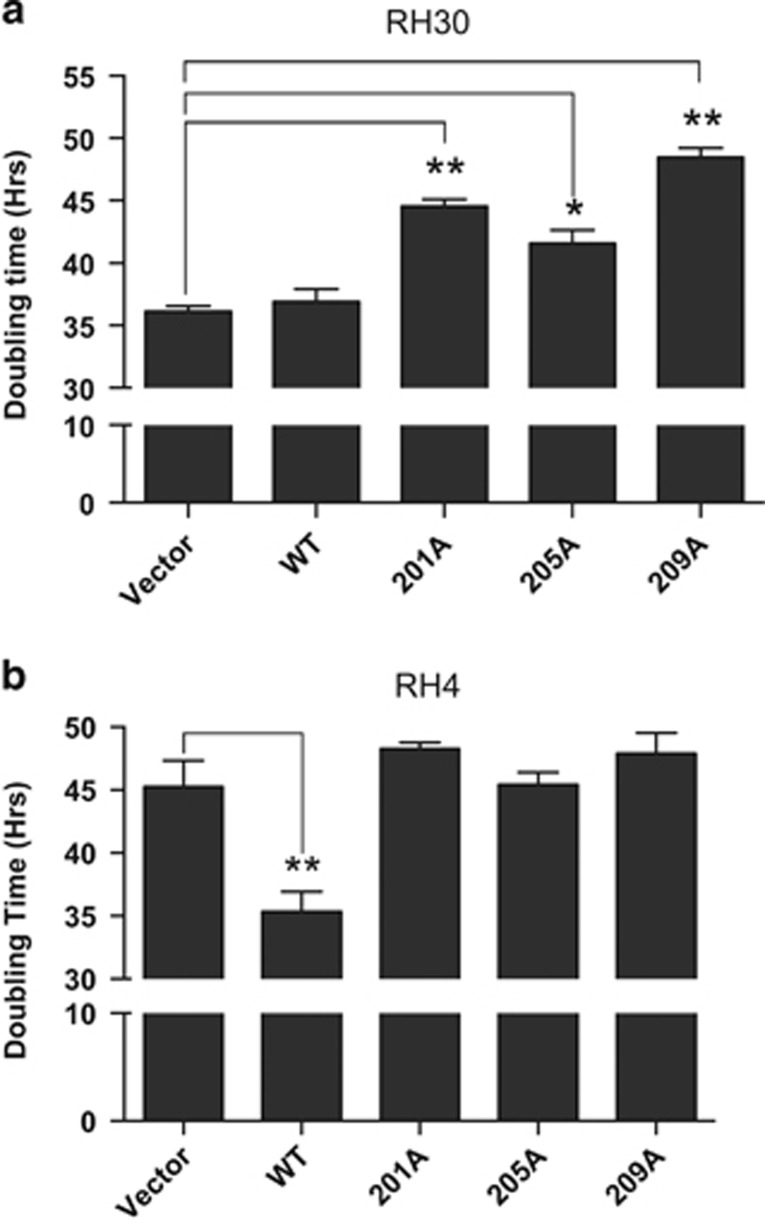
Effect of inhibiting PAX3-FOXO1 phosphorylation on ARMS tumor cell proliferation. (**a**) RH30 or (**b**) RH4 ARMS tumor were cells stably transduced with empty vector (vector), wild-type PAX3-FOXO1 (WT) or the indicated PAX3-FOXO1 phosphomutants, as described in the Materials and Methods. Cells were plated and allowed to grow for up to seven days. On each day the cell density was determined using the CCK-8 cell counting kit, densities were graphed as a function of time, and doubling times were determined using GraphPad Prism 6 software. Error bars represent the standard deviation from three independent determinations and *P*-values were computed using non-parametric two-way analyses of variance comparing each treatment condition to results seen with the empty vector transduced negative control. (*P=0.01, **P=0.003).

It was reported that inhibition of GSK3β decreased the viability of ARMS tumor cells.^19^ Similarly, we found that treatment of cells with GSK3β inhibitors for extended periods of time (>72 h) resulted in a level of cell death that precluded the use of these inhibitors in our proliferation assay (data not shown). Therefore, to determine the effect specifically inhibiting PAX3-FOXO1 phosphorylation has on proliferation, we used RH30 and RH4 cells stably transduced with PAX3-FOXO1 or the individual PAX3-FOXO1 phosphomutants. We saw no significant changes in the doubling time of RH30 cells stably transduced with wild-type PAX3-FOXO1 relative to the empty vector negative control. However, the stable expression of the phospho-incompetent S201A, S205A or S209A resulted in an increased doubling time from 35 h to nearly 50 h (Figure 4a). RH4 cells inherently have a slower doubling time relative to RH30 cells (45 h vs 35 h, respectively). The stable expression of PAX3-FOXO1 decreased the doubling time from 45 to 35 h relative to empty vector, a decrease that was lost upon the stable expression of any of the PAX3-FOXO1 phospho-incompetent mutants (Figure 4b).

### Inhibition of PAX3-FOXO1 phosphorylation reduces the anchorage-independent growth of ARMS tumor cells

**Figure 5:**
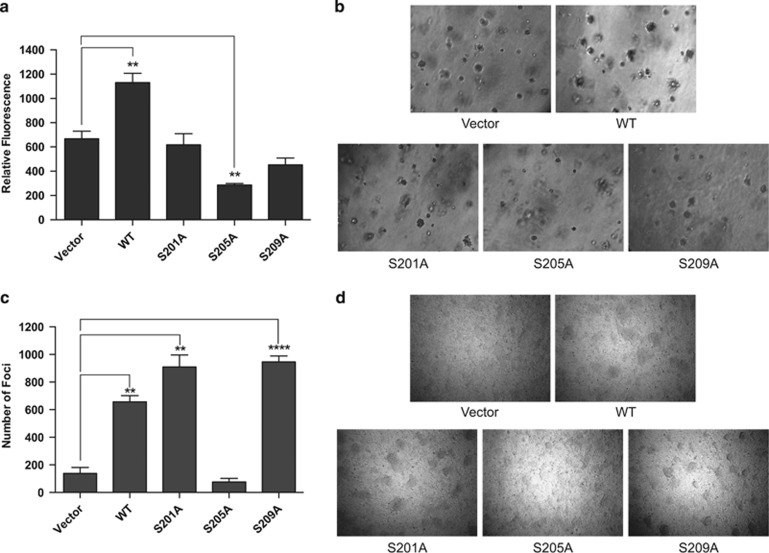
Effect of inhibiting PAX3-FOXO1 phosphorylation on anchorage-independent growth. (**a** and **b**) RH30 or (**c** and **d**) RH4 ARMS tumor cells were stably transduced with empty vector (vector), wild-type PAX3-FOXO1 (WT) or the indicated PAX3-FOXO1 phosphomutants. (**a**) RH30 cells were plated in soft agar, incubated for 1 week, and the extent of transformation was determined with a fluorescent assay, as described in the Materials and Methods. Results are presented as Relative Fluorescence, error bars represent the standard deviation from three independent determinations and *P*-values were computed using non-parametric two-way analyses of variance comparing each treatment condition to results seen with the empty vector-transduced negative control (**P=0.002). (**b**) Representative images of colony formation. (**c**) Cells were grown to confluency, after which they were allowed to grow an additional 5 days. Total number of colonies was counted, the results are presented as Number of Foci, error bars represent the standard deviation from four independent determinations, and *P*-values were computed using non-parametric two-way analyses of variance comparing each treatment condition to results seen with the empty vector transduced negative control (**P—0.0006, ****P<0.0001). (**d**) Representative phase contrast microscopy images of colony formation were taken.

The ability of cells to grow in the absence of a solid support is considered the benchmark for determining the transformation capacity of cells. Therefore, we determined how inhibition of PAX3-FOXO1 phosphorylation affects ARMS tumor cell anchorage-independent growth. As described above for the determination of proliferation, we were unable to utilize small molecule inhibitors in these experiments and therefore used cells stably expressing PAX3-FOXO1 phosphomutants. Further, RH4 cells are not amenable to soft agar assays,^25^ so we determined the anchorage-independent growth ability of these cells through focus-formation assays. We observed that the ectopic expression of wild-type PAX3-FOXO1 increased the ability to form colonies in soft agar and the ability to form foci for RH30 and RH4 cells, respectively (Figure 5). We found that inhibition of phosphorylation at Ser201 or Ser209 removed this PAX3-FOXO1-dependendent increased anchorage-independent growth with RH30 but not RH4 cells (Figure 5). In contrast, inhibition of phosphorylation at Ser205 significantly inhibited anchorage-independent growth in both cell lines (Figure 5).

### PAX3-FOXO1 is phosphorylated at Ser201 and Ser205 in human primary ARMS tumors and cells invading surrounding normal tissue

To date, studies examining the direct role that phosphorylation of PAX3-FOXO1 plays in myogenesis and ARMS tumor development have been predominantly limited to *in vitro* cellular systems^15, 16, 17, 19, 26^ and have yet to examine the presence and pattern of these phosphorylation events in primary tumor specimens. Therefore, we determined the presence of PAX3-FOXO1 phosphorylation in human patient primary tumors using affinity-purified antibodies specific for the fusion protein when phosphorylated at Ser201 or Ser205. These antibodies are highly specific for their antigenic target, as demonstrated by the presence of a single band on western blot analysis of total cellular extracts from multiple ARMS tumor cells, melanoma cell lines and primary myoblasts.^15, 16, 17, 27^ We initially utilized tumor tissue-derived from an ARMS primary tumor obtained from a right lung transbronchial biopsy from a 17-year-old male patient with the tumor testing positive for the t(2:13)(q35;q14) chromosomal translocation (data not shown). Although the presence of ARMS in pulmonary tissue is rare,^28, 29^ this sample provides the perfect internal negative control because the surrounding normal lung tissue does not express PAX3 or PAX3-FOXO1.

**Figure 6:**
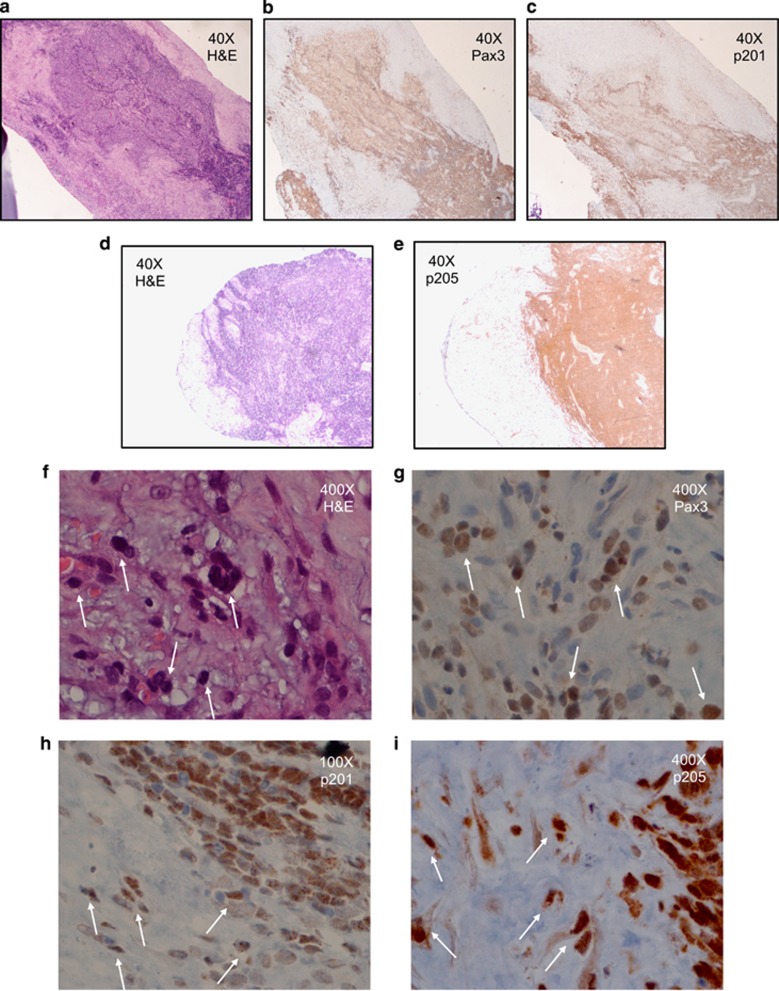
Expression of phosphorylated Pax3-FOXO1 in human primary tumor. Tumor sections were derived from a right lung transbronchial biopsy from a 17-year-old patient with a primary ARMS tumor testing positive for the t(2:13)(q35;q14) chromosomal translocation by FISH analysis. Sections were stained with hematoxylin and eosin (**a**, **d** and **f**), or antibodies that recognize Pax3 (**b** and **g**), Pax3 phosphorylated at Ser201 (**c** and **h**) or Pax3 phosphorylated at Ser205 (**e** and **i**) and magnifications are indicated in each panel.

We stained tissue with a Pax3-specific antibody, demonstrating the expression of PAX3-FOXO1 in ARMS primary tumor cells but not the surrounding normal tissue (Figure 6b). Using our Ser201 phospho-specific antibodies,^16^ we found a majority of the tumor stained positive for phosphorylation at Ser201 and that this staining is enriched around the periphery of the tumor (Figure 6c). Similarly, use of our Ser205 phospho-specific antibodies^17^ demonstrated that ARMS tumors also stain positive for phosphorylation at Ser205. However, unlike the Ser201 staining, the staining for phosphorylation at Ser205 is relatively strong and mostly uniform throughout the body of the tumor (Figure 6e). Finally, individual cells invading the surrounding normal tissue stain positive for phosphorylation at both Ser201 and Ser205 (Figures 6f–i). The results presented here are representative of multiple determinations (data not shown).

## Discussion

Present treatments for ARMS, which primarily include surgery, radiation and generalized chemotherapy, meet with little success due in part to a higher propensity for metastasis and resistance to treatment.^2, 30, 31^ One of the obstacles to finding better treatments is in elucidating druggable molecular mechanisms endemic to ARMS. Although several studies focused on manipulating downstream pathways effected by the oncogenic fusion protein PAX3-FOXO1,^32^ ongoing clinical trials demonstrate that these treatments are not as effective as originally hoped.^33, 34^ Further, although inhibiting downstream pathways is logical, these treatments do not target the defining genetic aberration, PAX3-FOXO1, which was shown to directly contribute to many ARMS tumor phenotypes.^35^ Therefore, directly targeting PAX3-FOXO1 is logical for the development of novel pharmaceutical therapies to treat ARMS.

The inhibition of phosphorylation through the use of small molecules is an intensely pursued line of investigation for potential cancer treatments.^36^ Although others described the effects of kinase inhibitors on PAX3-FOXO1 biological activities and ARMS tumor phenotypes,^18, 19^ neither of these studies provided direct evidence correlating these effects with changes in the phosphorylation of PAX3-FOXO1 at its known sites.^15, 16, 17^ Therefore, we examined how the direct inhibition of PAX3-FOXO1 phosphorylation affects the known ARMS tumor phenotypes of migration, invasion, proliferation and anchorage-independent growth. In this report, our results are the first to demonstrate that phosphorylation of PAX3-FOXO1, primarily at Ser201, is a valid molecular mechanism that contributes to the progression of ARMS.

We found that treatment of two ARMS cell lines with either a general or highly specific GSK3β inhibitor, which targets the kinase responsible for phosphorylating PAX3-FOXO1 at Ser201,^16^ resulted in titratable decreases in the phosphorylation of endogenous PAX3-FOXO1 at this site (Figure 1), which directly correlated to reductions in migration and invasion (Figures 2 and 3). Although compelling, these results alone are not definitive evidence, because GSK3β may affect other non-PAX3-FOXO1-related biological pathways, as was demonstrated for the translocation-negative embryonal rhabdomyosarcoma.^24^ However, we found that directly inhibiting phosphorylation at Ser201 through mutational analysis, which removes phosphorylation of the fusion protein independent of GSK3β action, reduces migration, invasion and proliferation (Figures 2, 3 and 4), and inhibiting phosphorylation at Ser205 reduces proliferation and anchorage-independent growth (Figures 4 and 5). Therefore, combined with the results of the small molecule inhibitors, our results allow us to conclude that phosphorylation of PAX3-FOXO1, primarily at Ser201 but with contributions by Ser205, is an important contributor to PAX3-FOXO1-dependent ARMS tumor phenotypes.

ARMS is a heterogeneous tumor^37^ and, as such, cell lines derived from patients exhibit differences in how they manifest tumor phenotypes.^38, 39^ Our results illustrate this fact, evidenced by our observed differences in proliferation, anchorage-independent growth and invasion between RH30 and RH4 cell lines (Figures 3, 4 and 5). However, we observed that the inhibition of phosphorylation of the fusion protein at Ser201 and Ser205 consistently inhibited these PAX3-FOXO1-dependent effects, regardless of the biological differences between cell types or their response to the ectopic expression of equivalent levels of PAX3-FOXO1. Further, even though these cells express endogenous PAX3-FOXO1, the ectopic expression of physiologically relevant levels of phospho-incompetent mutants is sufficient to inhibit tumor phenotypes. This fact suggests that non-phosphorylated PAX3-FOXO1 may act in a dominant-negative manner with respect to its phosphorylated counterpart. Taken together, our results support the idea that inhibition of PAX3-FOXO1 phosphorylation is a viable avenue for novel therapy development that could be applicable to translocation-positive ARMS tumors.

In addition to our *in vitro* evidence, it was important for us to validate the clinical relevance of these studies in primary patient samples. To date, the lack of phosphorylation-specific antibodies that were functional in immunohistocytochemistry made this difficult. However, the successful use of our phosphorylation-specific antibodies in immunohistocytochemistry allowed us to demonstrate the presence and pattern of PAX3-FOXO1 phosphorylation in primary tumor samples for the first time. Using rarely occurring primary translocation-positive ARMS tumor samples from a lung transbronchial biopsy,^28^ we found PAX3-FOXO1 is phosphorylated at Ser201 in primary ARMS tumor samples (Figures 6c and h). Consistent with our *in vitro results*, in which phosphorylation at Ser201 is important for tumor cell migration and invasion, we found staining for this event to be stronger around the periphery of the tumor (Figure 6c), where invading and migrating cells originate, and in cells that are actively infiltrating the surrounding normal tissue (Figure 6h).

We also found that PAX3-FOXO1 is phosphorylated at Ser205 in this same tumor sample (Figures 6e and i). Consistent with our *in vitro* results showing that phosphorylation at Ser205 contributes to proliferation, staining for this event is even throughout the tumor (Figure 6e), in direct contrast to staining for phosphorylation at Ser201. Further consistent with the contributions phosphorylation at Ser205 makes to anchorage-independent growth, a process essential for cells to establish a tumor at a distant site, we found intense staining for this event in cells invading normal tissue (Figure 6i). Taken together, these results show for the first time that PAX3-FOXO1 is phosphorylated at Ser201 and Ser205 in primary ARMS tumors, that these phosphorylation events are enriched in cells actively invading the surrounding normal tissue, and that they provide clinical validation for their *in vitro* effects on ARMS tumor phenotypes.

The results presented in this work support the idea that inhibiting phosphorylation of PAX3-FOXO1, particularly at Ser201, is an intriguing possibility for future ARMS therapies. Along these lines, GSK3β inhibitors are effective in reducing the growth and chemotherapeutic resistance of several non-ARMS cancer cells^40^ and suppresses growth and renewal of embryonal RMS tumors.^24^ Even more important for potential clinical applicability, small molecule inhibitors of GSK3β, such as lithium chloride and Tideglusib, were proven to be non-toxic in animal models and humans, and are useful for treating a wide range of cancers, including ovarian, colorectal, neuroblastoma and prostate.^41, 42, 43, 44, 45^

In conclusion, our data describe a molecular mechanism by which altering the posttranslational status of PAX3-FOXO1, in particular at Ser201, attenuates known ARMS oncogenic phenotypes. The *in vitro* results are supported through *in vivo* studies demonstrating the clinical relevance of these phosphorylation events. Taken together, these conclusions support the idea that use of small molecule inhibitors targeting GSK3β, molecules proven safe and effective in other unrelated cancers, may be a viable route for the development of novel therapies for ARMS through animal studies and pre-clinical trials.

## Materials and methods

### Cell lines and cell culture

The ARMS tumor cell line RH30 was purchased from the American Type Culture Collection. The 293T and ARMS tumor cell line RH4 were generous gifts from Dr. Gerard Grosveld. All cells were cultured in Dulbecco's Modified Eagle Media (supplemented with 10% FBS (HyClone Laboratories, Logan, UT, USA), penicillin G (200 U/ml) and streptomycin (200 μg/ml)) and grown in a humidified incubation chamber at 37 °C in 5% CO_2_.

### Construction of PAX3-FOXO1 expression vectors

PAX3-FOXO1 was cloned into the modified, low-copy number retroviral expression vector MSCV-IRES-Puro, which contains the backbone of the low copy pSMART GC LK vector (Lucigen, Middleton, WI, USA). We introduced a single nucleotide silent mutation, thereby creating a novel BamHI site in PAX3-FOXO1 that facilitated the cloning of phospho-incompetent mutants. The presence of the IRES allows the dual expression of PAX3-FOXO1 and the puromycin resistance gene as a single transcript, both under control of the constitutively active Murine Stem Cell Virus promoter.^46^ Phospho-specific point mutants in which the indicated serine was individually mutated to the phospho-incompetent alanine (S201A, S205A, S209A) were created by PCR amplification using previously created Pax3 point mutants as the template and primers containing a BamHI restriction sequence at the 3'-end. All constructs were confirmed by sequencing.

### Stable transduction of ARMS tumor cell lines

ARMS tumor cells were stably transduced with the empty vector, wild-type PAX3-FOXO1 or PAX3-FOXO1 phosphomutants (S201A, S205A or S209A) as previously described.^5, 17^ Retroviral stocks were generated by the transient transfection of 293T cells with 3 μg each of retroviral packaging vectors pSRα-G and p-EQ-PAM-E and 3 μg of the above-described expression vectors using the *TransIT*-LT1 reagent (Mirus, Madison, WI, USA). Culture supernatants containing virus were collected between 36 and 72 h after transfection, filtered (0.45 μm) and subsequently used for a single transduction, as previously described.^16, 17^ Two to 3 days post transduction, transduced cells were selected by incubation in media supplemented with puromycin (2.5 ng/ml) until complete cell death was observed in the non-transduced control cells.

### Antibodies and western blot analysis

Antibodies specific for PAX3-FOXO1 (anti-Pax3) or PAX3-FOXO1 phosphorylated at Ser201 (anti-Pax3[p201]) were produced as described previously.^17, 47^ Western blot analysis of endogenous PAX3-FOXO1: non-transduced RH30 or RH4 cells were grown to approximately 80% confluency. Growth media was replaced with media containing increasing concentrations of the GSK3β inhibitors LiCl (10 mM, 20 mM, or 40 mM) or AR-A014418 (2 μM, 10 μM, or 20 μM) and incubated for 24 h at 37 °C and 5% CO_2_. Alternatively, stably transduced cells were grown to 80% confluency in the absence of any inhibitors and harvested. Equal amounts of total cell lysates (25 μg) were separated by 8% SDS–PAGE and the analysis was performed as previously described.^15, 16^

### *In vitro* wound-healing assays

Non-transduced RH30 or RH4 cells were seeded onto a 24-well plate at 200 000 cells per well. Growth media was replaced with media containing increasing concentrations of LiCl (10 mM, 20 mM or 40 mM) or AR-A014418 (2 μM, 10 μM or 20 μM) and incubated for 24 h at 37 °C and 5% CO_2_. Alternatively, stably transduced RH30 or RH4 cells were seeded onto a 24-well plate at 300 000 cells per well. A 100 μl gel-loading tip was used to generate a scratch in the monolayer, the cells were carefully washed twice with 1 × phosphate-buffered saline to remove floating cells, and subsequently incubated with fresh media containing the GSK3β inhibitors or non-supplemented media, respectively. Images were captured at exact spots every 15 min for 6 h using an Olympus time-lapse microscope (Olympus, Pittsburgh, PA, USA) with subsequent analysis using SlideBook software. Sixty to 80 individual cells from four independent images were tracked and the velocity was determined for each cell using ImageJ software.

### Invasion assays

Non-transduced RH30 cells were grown on 100mm dishes until approximately 70% confluent, at which time they were incubated in the presence of GSK3β inhibitors at the concentrations described above. After 24 h, the cells were harvested and the invasive potential was determined using the BD Biocoat Tumor Invasion System (Becton Dickinson, East Rutherford, NJ, USA) as previously described^27^ using 100 000 cells suspended in media containing the respective inhibitors in the apical plate and proliferation media supplemented with hepatocyte growth factor (hHGF, PeproTech, Rocky Hill, NJ, USA) at 25 ng/ml and the respective inhibitors in the lower plate. The invasive potential of the stably transduced RH30 cells was determined as just described, except all incubations were performed in the absence of inhibitors. All samples were determined in triplicate and *P*-values were computed using non-parametric two-way analysis of variance.

### Proliferation assay

The proliferation rate of stably transduced RH30 and RH4 cells was assessed using a CCK-8 colorimetric assay kit (Cell Counting Kit-8, Dojindo Molecular Technologies, Rockville, MD, USA). Cells were seeded at a density of 25 000 cells per well in a 24-well plate and allowed to grow for up to 7 days. The assay was conducted in triplicate, with an independent well being used for each replicate for each day of cellular growth. On each day of growth, 50 μl of CCK-8 solution was added to the individual well being tested and incubated for 2 h in a humidified chamber at 37 **°**C in the presence of 5% CO_2_. Color formation was detected by measuring the absorbance at 450 nm using a microplate reader, plotted as a function of time, and doubling times calculated using GraphPad Prism 6 software. *P*-values were computed using non-parametric two-way analysis of variance.

In separate experiments, once reaching 100% confluence, stably transduced RH4 cells were allowed to grow an additional 5 days to determine their foci forming capacity. Total numbers of foci formed were counted, all samples were determined in quadruplicate, and *P*-values were computed using non-parametric two-way analyss of variance.

### Anchorage-independent growth in soft agar

Anchorage-independent growth assays were performed using a CytoSelect 96-well cell transformation assay kit (Cell Biolabs, Inc., San Diego, CA, USA). Stably transduced RH30 cells were plated in 1.2% agar in a 96-well plate at 5000 cells/well and cultured for 8 days at 37 °C and 5% CO_2_. Transformation and colony formation was determined using the CyQuant GR fluorescent dye according to the protocol provided by the manufacturer.

### Immunohistocytochemistry

Four micrometer-thick sections of human primary ARMS tumor were mounted on glass slides and incubated in an oven overnight at 65 °C to ensure tight adherence of tissue to the slides. Deparaffinization was carried out using xylene, graded alcohols and distilled H_2_O. The sections were loaded onto the Ventana Ultra IHC stainer (Ventana Medical Systems, Inc., Tuscon, AZ, USA) and prepared according to the manufacturer's specifications. Briefly, slides (tissues) were treated with Cell Conditioner (antigen retrieval solution) at 95 °C for 92 min. After rinsing with reaction buffer, the slides were warmed to 36 °C. I-View Inhibitor was applied for 4 min; the slides were rinsed using reaction buffer. The sections were then incubated with antibodies specific for Pax3 or Pax3 phosphorylated at Ser201 or Ser205 for 2 h at 36 °C. After incubation, all slides were treated with Ventana's Blocker A-B solution for 8 min. The slides were rinsed with reaction buffer; I-View Biotin Ig was applied for 8 min. After rinsing with reaction buffer, the slides were treated with I-View SA-HRP for 8 min, rinsed with reaction buffer. I-View DAB and I-View H202 was applied to the slides for 8 min after which the slides were rinsed in reaction buffer. The slides were treated with I-View Copper for 4 min, rinsed with reaction buffer, stained for 4 min with hematoxylin and toned with Bluing reagent for 4 min. Once the reactions were completed, the sections were washed in soapy distilled H_2_O, dehydrated (graded alcohols), cleared (xylene) and cover-slipped.

## Notes

Dr Hollenbach and Mr Miller are co-authors on US Patent #8,304,521, 'Phospho-specific anti-Pax3 antibodies' and are co-signees on licensing agreements with Abcam and Kerafast to market the phospho-specific antibodies described in this manuscript.
